# Liquid-Metal Enabled Droplet Circuits

**DOI:** 10.3390/mi9050218

**Published:** 2018-05-05

**Authors:** Yi Ren, Jing Liu

**Affiliations:** 1 Department of Biomedical Engineering, Tsinghua University, Beijing 100084, China; reny14@mails.tsinghua.edu.cn; 2 Technical Institute of Physics and Chemistry, Chinese Academy of Sciences, Beijing 100190, China; 3 School of Future Technology, University of Chinese Academy of Sciences, Beijing 100049, China

**Keywords:** droplet circuits, liquid metal, quantum tunneling effect, solution electronics, electron transport, ionic conduction, quantum computing, brain-like intelligence

## Abstract

Conventional electrical circuits are generally rigid in their components and working styles, which are not flexible and stretchable. As an alternative, liquid-metal-based soft electronics offer important opportunities for innovation in modern bioelectronics and electrical engineering. However, their operation in wet environments such as aqueous solution, biological tissue or allied subjects still encounters many technical challenges. Here, we propose a new conceptual electrical circuit, termed as droplet circuit, to fulfill the special needs described above. Such unconventional circuits are immersed in a solution and composed of liquid metal droplets, conductive ions or wires, such as carbon nanotubes. With specifically-designed topological or directional structures/patterns, the liquid-metal droplets composing the circuit can be discrete and disconnected from each other, while achieving the function of electron transport through conductive routes or the quantum tunneling effect. The conductive wires serve as electron transfer stations when the distance between two separate liquid-metal droplets is far beyond that which quantum tunneling effects can support. The unique advantage of the current droplet circuit lies in the fact that it allows parallel electron transport, high flexibility, self-healing, regulation and multi-point connectivity without needing to worry about the circuit break. This would extend the category of classical electrical circuits into newly emerging areas like realizing room temperature quantum computing, making brain-like intelligence or nerve–machine interface electronics, etc. The mechanisms and potential scientific issues of the droplet circuits are interpreted and future prospects in this direction are outlined.

## 1. Introduction

**Figure 1:**
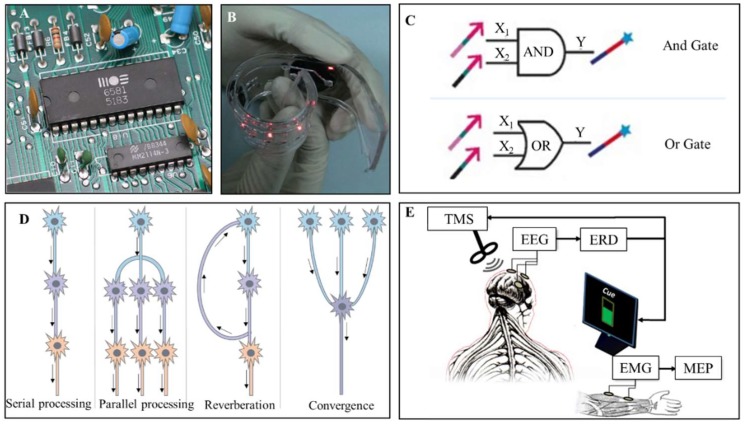
Respective kinds of electrical circuits. (**A**) Traditional rigid print circuit boards (PCB) circuits (https://upload.wikimedia.org/wikipedia/commons/0/0f/MOS6581_chtaube061229.jpg); (**B**) Soft electronics. Reproduced with permission from [8]; (**C**) Logic AND Gate and OR Gate based on DNA. Reproduced with permission from [6]; (**D**) Several typical types of neural circuits; (**E**) Brain–computer interface. Reproduced with permission from [9]. Note: TMS: transcranial magnetic stimulation; ERD: event-related desynchronization; MEP: motor-evoked potential; EEG: electroencephalogram; EMG: electromyogram.

Since the origin of electricity, it has become a necessity in daily life. Generally speaking, electrical circuits are rigid and continuous in their structures and components. Print circuit boards (PCB) have been commonly used in various situations (Figure 1A). However, classic rigid circuits cannot easily adapt to the human body due to poor flexibility and biocompatibility, limiting their value in the biomedical and health care fields. The increasing advancement of wearable devices and implantable systems has led to significant growth in flexible electronics (Figure 1B) [1]. Polymer nanomaterials, silk fibroin and liquid metal are being gradually adopted in soft electronics. Another main development trend in artificial circuits is molecular electronics. This was first proposed in 1974 by Aviram and Ratner [2], and refers to a field that seeks to fabricate electrical devices and circuits with single molecules and molecular monolayers [3,4,5,6]. The fabrication of molecular electrical devices includes single-molecule break junctions and molecular monolayer devices [3]. Tailored by chemical design and synthesis, the function of molecular components can be rather diverse. Deoxyribonucleic acid (DNA) has potential for molecular devices for its unique structure [5]. Figure 1C presents the logic ‘AND’ and ‘OR’ gate fabricated by DNA. Until now, molecular components including diodes, switches, memory and transistors have been intensively researched [6]. Those components can be combined to construct molecular-scale electronic computers [7]. Based on the electrical properties of the molecular diode switches, quantum mechanical calculations can also possibly be performed.

Besides these artificial circuits, electrical circuits in fact intrinsically exist throughout the human body, as shown in Figure 1D. For instance, Dejean et al. recently studied the neural circuits and cell types that mediate conditioned fear expression and recovery [8]. Moreover, voltage-gated channels are important switches for signal transportation in the central and peripheral neural systems [10]. Keeping the above facts in mind and without losing generality, we can divide electrical circuits into two main categories: biologically-inspired natural circuits and human-made artificial circuits. One more trend, as indicated in Figure 1E, is that a new category is emerging to combine naturally occurring neural circuits and artificial circuits to carry out complex functions. The core of such circuits can generally be called brain–computer interfaces (BCIs) [11,12,13,14]. Clearly, BCIs are significant for patients with serious disabilities such as tetraplegia and stroke. They especially mean a lot for future human needs in extending the limits of biological capability. At this stage, BCIs based on rapid serial visual presentation have already been used to detect and recognize objects, providing a viable approach to prompt human–machine systems [14].

Despite the widespread application of currently available circuits, they all unavoidably encounter the possibility of circuit break, which severely affects the normal operation of devices. As a remedy, we here propose an unconventional concept for electrical circuits to tackle the above challenges, which we call droplet circuits. Such an electrical circuit is enabled from liquid-metal droplets (LMDs) and is conductive in discontinuous form. The structure and working style of this kind of circuit are highly analogous and similar to that of the neuro-network system. Therefore it is expected to be very useful in innovating newly emerging areas such as room temperature quantum computing, brain-like intelligence, and brain–machine interfaces, etc.

**Figure 2:**
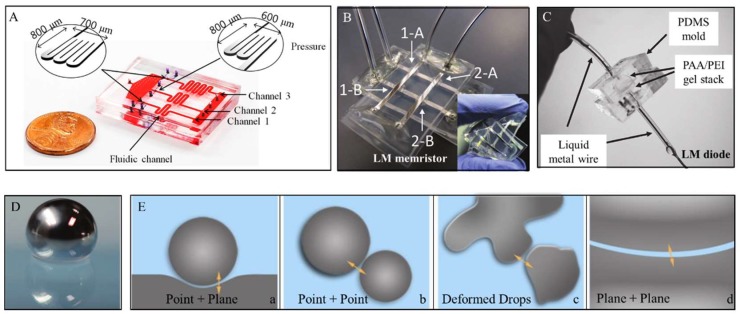
(**A**) Liquid-metal pressure sensor [16]; (**B**) A 2 × 2 crossbar array liquid metal memristor: 1-A, 1-B, 2-A and 2-B refer to the four bars respectively. Reproduced with permission from [18]; (**C**) Liquid-metal diode. Reproduced with permission from [19]; (**D**) Liquid-metal droplet. Reproduced with permission from [28]; (**E**) Four configurations of all-soft liquid metal quantum devices based on tunneling effect. Reproduced with permission from [31].

Liquid metal refers to alloys or metals with a low melting point, which can maintain their liquid phase around room temperature [15]. The electrical properties and biocompatibility of liquid metal have been proven in Yi et al.’s work [15]. In contrast to mercury, which is highly toxic, liquid metal based on gallium is relatively safe for medical applications. Liquid metal has recently been introduced into soft electronics and the biomedical field [15]. Liquid-metal sensors [16,17], memristors [18], diodes [19] and electrodes [20,21,22] have already been proposed for health monitoring and disease treatment (Figure 2A–C). Recently, some researches has been devoted to the study of liquid-metal droplets (Figure 2D), which show great potential in self-powered devices [23,24] and phagocytosis [25]. Yang et al. introduced millimeter-scale LMDs as thermal switches, unlocking new possible solutions for thermal management [26]. Tang et al. electrically controlled the size and rate of LMD formation [27]. Others fabricated non-stick LMDs by coating polytetrafluoroethylene particles [28] or graphene [29] on NaOH-treated LMDs. Chen et al. found that graphene-coated LMDs can be used as droplet-based floating electrodes [29]. Sivan et al. coated LMDs with n-type and p-type semiconducting nanopowders to study their electronic properties and electrochemical properties [30].

In contrast to previous studies, where all LMDs were contacted to realize electrical conduction, droplet circuits mean that the circuit is composed of discrete LMDs and can operate well, in the same way as traditional circuits. For this purpose, we would speculate that when the gap between the two LMDs is small enough, the electrical signal can be transported even though they are not connected. This assumption is dependent on the quantum tunneling effect theory, which is a physical phenomenon in which a micro-particle such as an electron can tunnel through a barrier that it classically could not surmount. Thus, droplet circuits can transfer electrical signals even though LMDs are separated in space. Zhao et al. have proposed a transformable soft quantum device based on liquid metal [31]. They found that liquid metal droplets can be adopted to create tunnel junctions and defined four configurations of all-soft quantum devices, as shown in Figure 2E. Based on their research, this article further explores the potential of liquid-metal droplets in constructing droplet circuits and preliminarily demonstrate its probability. Compared to traditional rigid or soft circuits, liquid-metal droplet circuits provide more flexibility without the problem of circuit breaks.

## 2. Electrical Conduction via the Quantum Tunneling Effect

The quantum tunneling phenomenon cannot be explained by classical mechanics, as it occurs only at the quantum scale. Compared to classical mechanics, matter in quantum mechanics has the properties of waves and particles, involving the Heisenberg uncertainty principle [32,33,34]. The Heisenberg uncertainty principle states that one can never exactly know the position and speed of a particle at the same time. Thus, events which seem to be impossible in classical mechanics become possible in quantum mechanics. This can be used to explain the quantum tunneling phenomenon.

**Figure 3:**
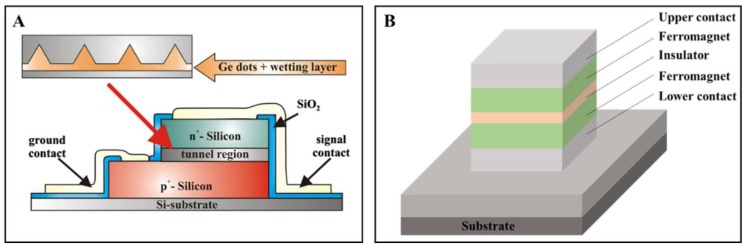
Illustration of the quantum tunneling effect and conventional typical application devices. (**A**) A Ge quantum dots interband tunneling diode. Reproduced with permission from [36]; (**B**) Diagram of a ferromagnet tunnel junction.

Quantum tunneling is essential on many occasions, including nuclear fusion in the sun [35], astrochemistry in interstellar clouds, quantum biology, tunnel diodes (Figure 3A) [36], tunnel junctions (Figure 3B) [37], scanning tunneling microscope and quantum computing, etc. A tunnel junction is where two conductors are separated by a thin insulator to create a simple barrier between them. It can be applied to measure voltages and magnetic fields.

## 3. Fabrication of LMDs and Composition of Droplet Circuits

**Figure 4:**
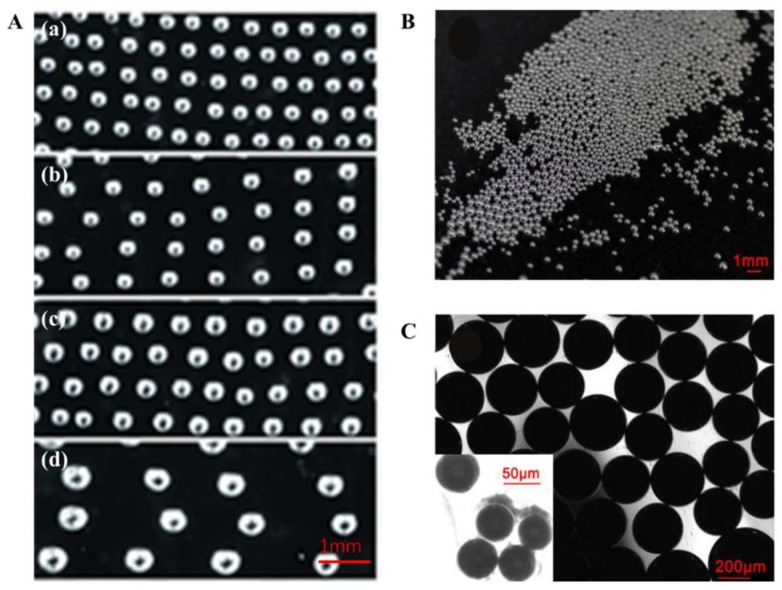
(**A**) Liquid-metal droplets (LMDs) floating in hydrogel with varied diameters; (**a**) 60 μm, (**b**) 90 μm, (**c**) 160 μm, (**d**) 210 μm. Reproduced with permission from [38]; (**B**) LMDs assembly fabricated through injection way. Reproduced with permission from [39]; (**C**) Microscopic image showing a single layer of liquid metal micro-droplets closely sitting together with thin interface between each other. Reproduced with permission from [39].

Until now, the methods for the fabrication of LMDs have been relatively diverse and easily available, including methods like sonication, molding, and flow-focusing [27]. Recently, Yu et al. created a method named suspension 3D printing [38], which can help quickly realize three various dimensional droplet patterns. They successfully patterned LMDs into a self-healing hydrogel (Figure 4A) and studied the relationship between the process parameters, supporting gel concentration, and the deposited micro-droplet geometry. Before that, the present lab also successfully prepared LMDs on a large scale through a fabrication method that does not use channels [39], as shown in Figure 4B. The microscopic image (Figure 4C) presents a single layer of liquid-metal micro-droplets, which were sitting together with a thin interface between each other. From the insert of Figure 4C, we can see that although the distance between the LMDs is extremely small, they are in fact discrete. Furthermore, Tian et al. proposed a microfluidic chip for liquid-metal droplet generation and sorting. Their system could manipulate these neutral liquid-metal droplets in nonconductive fluid [40]. All those studies provide viable methods for the fabrication of LMDs and the construction of droplet circuits, and more efforts should be made to improve the technology.

A rising trend in liquid metal is to cover LMDs with other materials to fabricate multifunctional circuits. For instance, liquid metal can be sealed into polymers to maintain its pattern. Water films and nanoparticles are applied to prevent the formation of an oxide layer, hold the stability and keep them discrete from each other [28,41]. Moreover, by coating LMDs with magnetic particles, they can be induced to move with the external magnetic field. Besides magnetic control, an electrical field can also induce the movement of liquid metal. Therefore, by coating specific materials, we can fabricate liquid-metal droplets with the required properties.

## 4. Droplet Circuits in Solution

### 4.1. Mechanism of Liquid-Metal-Based Droplet Circuits

Droplet circuits are mainly composed of discontinuous LMDs, ions and conductive wires such as carbon nanotubes and operate in electrolyte solution. LMDs and carbon nanotubes are mixed up and cooperate to connect the circuit electrically. Carbon nanotubes have unique electrical, thermal and chemical properties, showing great potential in nanoelectronics [42], especially for transistor applications, owing to their benign carrier mobility and velocity. Therefore, carbon nanotubes can be selected to connect droplet circuits.

**Figure 5:**
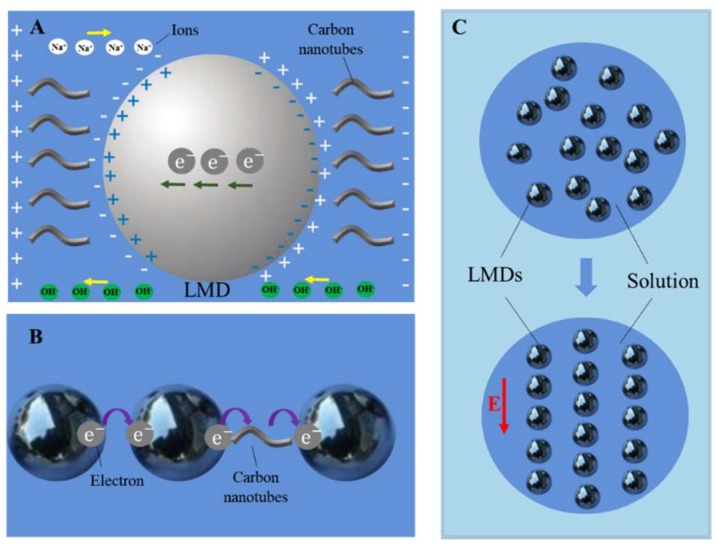
(**A**) The electrical environment of a LMD in NaOH solution: A LMD is surrounded by ions and conductive carbon nanotubes; tunneling effect between two LMDs; (**B**) Combination of two methods of communicating for LMDs in a circuit; (**C**) LMDs arranged with and without voltage.

Figure 5A shows the electrical conduction of droplet circuits. LMDs in droplet circuits are surrounded by carbon nanotubes and ions and electrons transfer along the pattern of LMDs with the assistance of carbon nanotubes and ions. There are two ways for LMDs to communicate in electrolyte solution, as shown in Figure 5B. When two LMDs are sitting closely enough, a quantum tunneling effect will happen and electrons can possibly transfer from one to another. If the distance of two LMDs becomes a little farther than the tunneling effect can support, carbon nanotubes and ions floating between them serve as transfer stations for electrons to flow. Through the quantum tunneling effect, carbon-nanotube transfer stations and ionic routes, the discrete LMDs are electrically connected and the whole circuit can work well. In general, LMDs are randomly arranged in electrolyte solution. If voltage is applied to the LMDs, they will be organized in order and connect the circuit (Figure 5C).

As the resistance of LMDs is smaller than other regions in electrolyte solution, the current will regularly transfer mainly along LMDs. In addition, one can change the pattern of LMDs to control the conducting direction of the current. That is, given a specific topological or directional design, the liquid-metal droplets composing the circuit can achieve the desired or regulative functions of electron transport through conductive routes or the quantum tunneling effect. Some newly emerging needs, such as room temperature computing or brain-like chips, can possibly be enabled based on such unconventional electrical circuits.

### 4.2. Configuration of the Liquid-Metal Droplet Circuit

#### 4.2.1. Low-Dimensional Droplet Circuits

**Figure 6:**
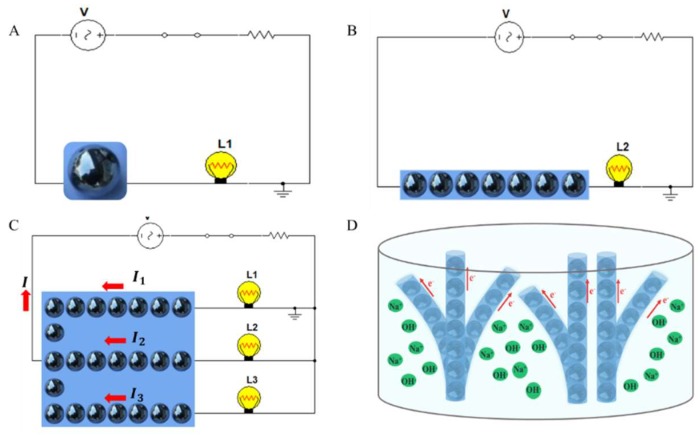
(**A**) Zero-dimensional (0D) droplet circuit; (**B**) 1 D droplet circuit; (**C**) 2 D droplet circuit; (**D**) Topological structure of droplet circuit.

Zero-dimensional (0D) droplet circuits refer to the case where only one LMD works. Figure 6A shows a schematic of a 0D circuit. The LMD is immersed in NaOH solution and replaces part of the wire. When the switch is closed, the lamp can be lit.

By increasing the number of LMDs and arranging them in a line, a one-dimensional (1D) droplet circuit can be formed. The line of LMDs can be inserted into a traditional circuit as an alternative to the original wire, as shown in Figure 6B. Similarly, one can arrange LMDs in a plane with a specific pattern to realize a two-dimensional (2D) droplet circuit (Figure 6C). In principle, this circuit should also satisfy Kirchhoff’s current law:

(1) $$\sum I_{in}=\sum I_{out}$$

Here, $I_{in}$ is the current flowing into a node and $I_{out}$ is the current flowing out from the node.

Kirchhoff’s law states that the charge input at a node is equal to the charge output. Therefore, the currents of the circuit in Figure 6C should meet the following equation:

(2) $$I=I_{1}+I_{2}+I_{3}$$

Due to existence of the electrolyte in the solution, charge loss is inevitable. The main loss pathways include the redox chemistry between NaOH and LMDs and the contact between NaOH and carbon nanotubes. However, these are small enough to be neglected since the conductivity of NaOH is much lower than LMDs and carbon nanotubes.

Furthermore, LMDs can be arranged in a special topological structure to fabricate three-dimensional (3D) or even dynamically transformable droplet circuits, as shown in Figure 6D. To form specific structures of 3D droplet circuits, polymer can be applied. LMDs can be sealed in polymer tubes and be arranged as required. Electrical or magnetic fields can be utilized at the same time. These 3D droplet circuits are flexible and able to transport electrons in the desired direction. Topological droplet circuits perfectly imitate the connection between neurons, which is beneficial to artificial neural connections.

The combination of liquid-metal and artificial circuits has been demonstrated before by Zhang et al. [43]. They injected liquid metal in the left and right side of the sciatic nerve near the femur of the interceptive lower part of a bullfrog body. It was found that the two electrodes successfully conducted the electrical stimuli signals to the nerves, which proves the feasibility of applying liquid metal to electrical circuits.

#### 4.2.2. High-Dimensional Droplet Circuits

**Figure 7:**
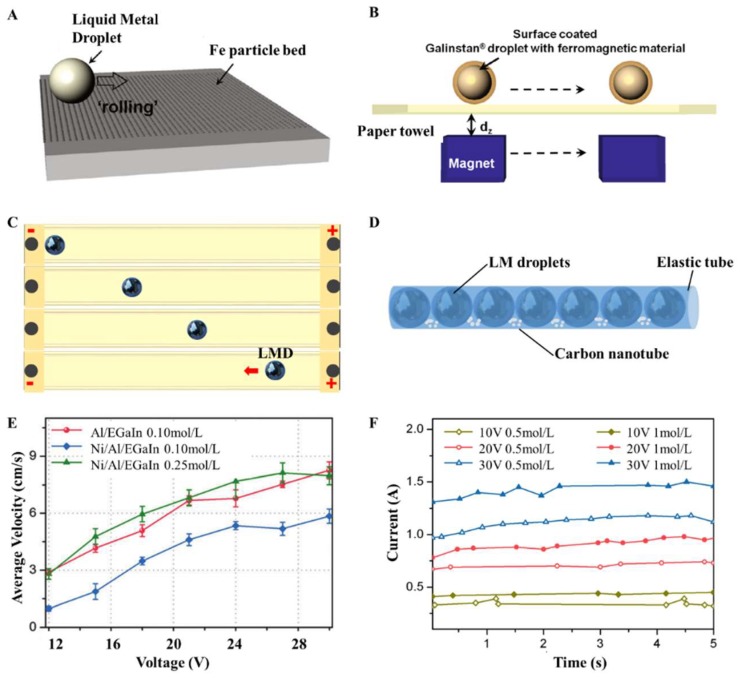
(**A**) Fabrication of Fe-coated LMDs. Reproduced with permission from [44]; (**B**) Surface-coated LMD with ferromagnetic materials induced by magnetic field. Reproduced with permission from [44]; (**C**) Sequential photos of LMD motion in an electrical field; (**D**) Liquid-metal droplet wire; (**E**) Average velocity of LMDs in a straight channel containing NaOH solution under exposure to different voltages, and the concentrations of the NaOH solution [46] (Reproduced with permission); (**F**) The electric current in response to different voltages, and the concentrations of the NaOH solution. Reproduced with permission from [47].

The further development of droplet circuits may be in more than three dimensions. In this regard, LMDs can be encapsulated into elastic tubes together with carbon nanotubes and electrolyte solution together to replace conventional rigid metal wires, as shown in Figure 7D. Moreover, since the pattern of LMDs can be changed by external magnetic or electrical fields, the circuit will become more fantastic and dynamically change over the time. Applying electrical or magnetic fields to LMD circuits, the structure of the circuits can be changed as required. For instance, LMDs can be induced to move by magnetic field when coating them with ferromagnetic materials (Figure 7A,B) [44]. With the help of aluminum, one can realize reliable motion control of the liquid metal droplets in the electrical field [45]. Figure 7C presents the sequential movement of a liquid-metal droplet propelled by an external electrical field. The velocity of Al/EGaIn and Ni/Al/EGaIn droplets in NaOH solution under different voltages was measured and calculated, as shown in Figure 7E [46]. Al provides a fuel source for LMDs to move, and they observed that Ni is not favorable for the electrical control of LMDs. It is apparent that the LMDs speed up with the increase of the voltage. Moreover, the current of the droplet circuits composed of LMDs in NaOH solution was relatively stable under constant voltages (Figure 7F), demonstrating promising applications of droplet circuits [47]. Recently, Isabela et al. fabricated a magnetocaloric ferrofluid based on Ga liquid metal [48]. They suspend gadolinium nanoparticles in a liquid gallium alloy and found that the material exhibited spontaneous magnetization and a large magnetocaloric effect. This is a significant progress in the magnetic control of liquid metal.

## 5. Discussion

This article exploits the basic features of droplet circuits and evaluates the probability of functional circuits enabled by liquid-metal droplets. Obviously, droplet circuits display unique advantages that may not be easily offered by conventional strategies. First of all, droplet circuits show a highly parallel electrical transporting capability. In addition, such circuits have excellent flexibility, self-healing capabilities and are stretchable. In contrast to existing liquid-metal soft electronics, LMD circuits consist of spatially transformable discrete liquid-metal droplets. It can tolerate greater elastic deformation and can be shaped into various electrical patterns.

Further, droplet circuits are fault-tolerant and self-error-correcting. Since LMDs can transport electrons while they are disconnected from each other, droplet circuits may not face circuit break errors. Even though the distance between LMDs can be changed by external factors such as strain and twist, carbon nanotubes will keep the circuit electrically connected. It is apparent that droplet circuits appear more robotic than traditional circuits, which is very much like the working style of a biological neurocircuit. The electrical resistance of liquid-metal droplet circuits may be higher due to the discontinuity and cross-talk between individual droplet chains, but since the quantum tunneling effect occurs within an extremely small space, the running time of the whole circuit will be greatly reduced. Particularly, droplet circuits can run in the wet environment of electrolyte solution. Electrical conduction involves not only LMDs and carbon nanotubes, but also ionic conduction. Ions in electrolyte solution play the role of electron transportation along with carbon nanotubes when the quantum tunneling effect of LMDs does not work.

Last but not least, the fabrication of liquid-metal-based droplet circuits is overall not complicated, since LMDs self-assemble in the environment of magnetic or electrical fields. The circuit configurations are in dynamic change at the same time.

**Figure 8:**
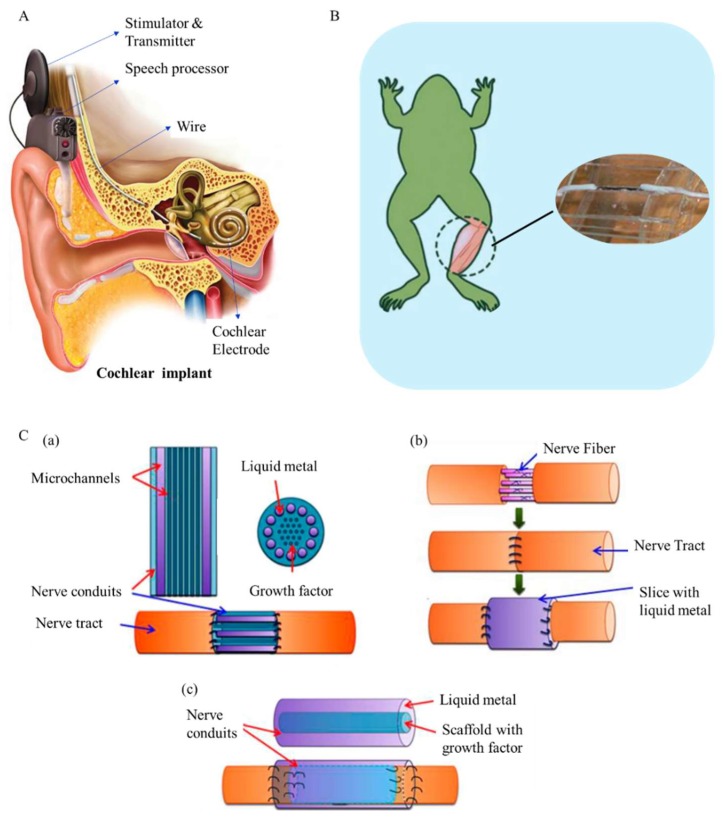
(**A**) The structure of a cochlear implant (https://upload.wikimedia.org/ wikipedia/commons/-f/f1/Electric-Acoustic-Stimulation-EAS.png); (**B**) The transected sciatic nerve of a bullfrog reconnected by liquid metal [49]; (**C**) Three kinds of nerve conduits to repair the injured peripheral nerve: Nerve conduit with microchannels (**a**), a thin slice (**b**) and concentric tubes (**c**) [49]. (Note: Figures reproduced with permission).

From a practical point of view, due to its unique advantages, such as high flexibility and benign electrical conductivity, liquid-metal-based droplet circuits can possibly be applied to make artificial retina or cochlea as an alternative to conventional rigid wires and electrodes. Figure 8A is the presentation of a cochlear implant. The wires and electrodes should be soft and biocompatible enough to ensure minimal damage to biological tissues. Liquid-metal-enabled droplet circuits could perfectly meet these requirements. Nerve repair and neural connection is another promising development for such droplet circuits. Efforts have been made to explore the possibility of liquid-metal neural restoration. Zhang et al. employed liquid metal to reconnect the transected sciatic nerve of a bullfrog (Figure 8B) and found that the measured electroneurographic signals under electric stimulation were similar to those from the intact sciatic nerves [49]. They further put forward three types of nerve conduits to restore damaged peripheral nerves, as shown in Figure 8C. Nerve conduits combined with liquid metal can take the form of microchannels, thin slices or concentric tubes. Unlike the former trial, the current droplet circuit offers more electron transport channels, such as electrically conductive liquid metal, ionic conduction and nanowires, etc. Therefore, it would better serve medical needs. Another potential that is worth of mentioning of droplet circuits lies in its role in constructing computing chips or devices that are different from the classical framework. With discrete LMDs, droplet circuits offer opportunities to perform as quantum processors or to carry out quantum calculating, which may help the design of future quantum computers.

## 6. Conclusions

This work presents a new conceptual droplet circuit that is enabled by liquid metal and demonstrates its potential role in the electronics field. The advantage of droplet circuits lies in that they address well the problem of circuit breaks and allow more flexibility and self-healing features, and show great potential for the development of smart soft electronics, which could imitate well the biological neurocircuits in nature. In addition, droplet circuits show potential for possible applications in future quantum calculation. Such droplet circuits are easily fabricated and self-error-correcting. This shows their promising value for the molding of large-scale application technologies. Further research could be conducted to fabricate stable and nano-scale liquid metal droplets in electrolyte solution and test the electrical properties of those droplets. The main difficulty of liquid-metal droplet circuits is the size of LMDs, which limits the progress of the liquid metal. Micro-scale and nano-scale circuits are currently the main trend. Compared to complementary metal oxide semiconductor (COMS) fabrication technology, the fabrication of liquid metal requires more improvement. Clearly, science, technology and applications in this direction require systematic investigation and integration in the future.
